# Real-world bleeding risk and molecular interaction profiles of direct oral anticoagulants: a comparative study integrating clinical outcomes and *in silico* docking

**DOI:** 10.3389/fphar.2026.1828307

**Published:** 2026-06-05

**Authors:** Lutfi Cagatay Onar, Ersin Guner, Ibrahim Yilmaz

**Affiliations:** 1 Department of Cardiovascular Surgery, Dr. Ismail Fehmi Cumalioglu City Hospital, Ministry of Health, Republic of Turkey, Tekirdag, Türkiye; 2 Department of Pharmacy, Konya Numune Hospital, Ministry of Health, Republic of Turkey, Konya, Türkiye; 3 Unit of Pharmacovigilance, Dr. Ismail Fehmi Cumalioglu City Hospital, Ministry of Health, Republic of Turkey, Tekirdag, Türkiye

**Keywords:** bleeding risk, direct oral anticoagulants, factor Xa inhibitors, molecular docking, precision anticoagulation, real-world data, thrombin inhibitor

## Abstract

**Background:**

Differences in bleeding risk among direct oral anticoagulants (DOACs) may reflect not only exposure-related factors but also molecule-specific drug–target interaction characteristics. This study aimed to evaluate real-world bleeding outcomes and to complement these findings with molecular docking analyses as supportive, hypothesis-generating mechanistic context for safety differences among factor Xa inhibitors and a direct thrombin inhibitor.

**Methods:**

A retrospective cohort of 5,721 patients treated with apixaban, rivaroxaban, edoxaban, or dabigatran was analyzed using real-world clinical data. Demographic characteristics, comorbidities, non–life-threatening complications, life-threatening hemorrhagic events, and overall bleeding outcomes were systematically compared. Multivariable logistic regression modeling was used to identify independent predictors of bleeding, with apixaban as the reference agent. Molecular docking analyses were performed using AutoDock 4.2.6 to assess comparative binding energy profiles, hydrogen bond interactions, and root-mean-square deviation values between each DOAC and factor Xa or thrombin.

**Results:**

Life-threatening bleeding occurred in 3.1% of patients, while any bleeding event was observed in 13.5% of the cohort. Compared with apixaban, rivaroxaban (OR 1.52), dabigatran (OR 1.29), and edoxaban (OR 1.44) were independently associated with higher odds of bleeding. Advanced age and renal dysfunction were identified as additional independent predictors. Docking analyses indicated that apixaban exhibited the lowest docking-derived binding energy toward factor Xa (ΔG = −14.46 kcal/mol), whereas dabigatran showed selective interaction with thrombin (ΔG = −11.91 kcal/mol). These molecular findings are exploratory and hypothesis-generating and should be interpreted as supportive mechanistic context rather than direct predictors of clinical outcomes.

**Conclusion:**

In this retrospective cohort, apixaban was associated with a comparatively lower observed bleeding risk compared with other DOAC agents. Docking findings provide exploratory, hypothesis-generating mechanistic context that may help to contextualize observed clinical patterns.

## Introduction

Oral anticoagulants exert their effects through pharmacological modulation of key enzymatic steps in the coagulation cascade, and non-vitamin K antagonist oral anticoagulants (NOACs), also referred to as direct oral anticoagulants (DOACs), represent a significant evolution in anticoagulant direct factor Xa or thrombin inhibition (Jannati et al., 2024). While direct factor Xa inhibitors (apixaban, rivaroxaban, and edoxaban) target a critical upstream step in thrombin generation (Weitz and Bates, 2005), the direct thrombin inhibitor dabigatran inhibits fibrin formation by directly binding to the active site of thrombin (Dólleman et al., 2022). These agents differ substantially in molecular structure, target protease interaction profiles, and pharmacokinetic properties, and these differences may contribute to heterogeneity in clinical outcomes.

The principal adverse effect of DOAC therapy is bleeding, and this risk is determined not only by the degree of anticoagulant effect but also by the pharmacokinetic and pharmacodynamic, and drug-target interaction characteristics (Hindley et al., 2023). Pharmacodynamic variability in drug–target interactions, together with pharmacokinetic and patient-specific factors such as renal elimination, protein binding, and drug–drug interactions, contributes to heterogeneity in DOAC safety profiles.

Over the past decade, real-world observational data have progressively demonstrated that direct oral anticoagulants exhibit molecule-specific differences in bleeding and safety outcomes (Grymonprez et al., 2025). While historically classified as NOACs, the field has increasingly adopted the term DOACs, a designation that emphasizes their mechanism of direct factor Xa or thrombin inhibition.

A Belgian national cohort study reported that DOACs were associated with more favorable bleeding outcomes than vitamin K antagonists (VKAs), including lower rates of major and clinically relevant non-major bleeding as well as intracranial hemorrhage. In that study, apixaban and dabigatran were associated with lower risks of major or clinically relevant bleeding compared with rivaroxaban and edoxaban (Grymonprez et al., 2023). However, an analysis of the U.S. Food and Drug Administration Adverse Event Reporting System database reported higher rates of major and intracranial hemorrhage with edoxaban than with other DOACs, whereas apixaban and rivaroxaban demonstrated comparatively lower reporting rates. Edoxaban exhibited the highest proportional reporting ratio (PRR) for major bleeding (Qian et al., 2024; Wu et al., 2022). These observations highlight the importance of continued real-world safety evaluation of individual DOAC agents.

At the molecular level, efforts to interpret clinical safety differences have increasingly incorporated *in silico* modeling methods that characterize drug-target interactions at atomic resolution. Molecular docking and computer-aided modeling offer the possibility of generating comparative structural parameters such as docking-derived binding energy estimates, hydrogen bond networks, and interaction geometry. These molecular findings are presented in a clinically interpretable framework, aiming to support clinicians’ understanding of molecule-specific safety variability rather than to introduce molecular complexity into routine clinical decision-making.

Docking-derived binding energies should not be interpreted as direct surrogates of experimentally measured thermodynamic constants or binding affinity parameters. Rather, they offer a comparative structural framework for understanding molecule-specific pharmacodynamic interaction patterns. While these *in silico* approaches provide atomic-level mechanistic perspective, they do not capture *in vivo* pharmacokinetic variability and therefore serve primarily as hypothesis-generating tools rather than clinical predictors.

Therefore, the aim of this study was to evaluate the comparative safety profiles of direct factor Xa inhibitors (apixaban, rivaroxaban, and edoxaban) and the direct thrombin inhibitor dabigatran using real-world bleeding outcomes, complemented by molecular docking analyses as supportive mechanistic context. By examining real-world bleeding patterns alongside structural interaction characteristics between each drug and its coagulation target, we sought to clarify molecule-specific safety differences among DOAC agents in routine clinical practice. Previous studies have generally evaluated clinical outcomes or molecular characteristics separately, whereas approaches combining observed bleeding phenotypes with *in silico* modeling remain limited. By bringing these domains together, our study offers a clinically grounded, hypothesis-generating perspective that may help contextualize molecule-specific safety variability beyond what has been described in earlier investigations.

## Materials and methods

### Study design and population

This was a retrospective observational cohort study conducted in a tertiary referral center using real-world clinical data. All adult patients (≥18 years) who received one of the four DOACs-dabigatran, rivaroxaban, apixaban, or edoxaban—between January 2016 and December 2025 were screened. A total of 6,482 patients were initially identified during the screening process. Of these, 421 were excluded due to treatment duration <3 months, 167 due to incomplete clinical follow-up data, 94 due to documented poor medication adherence (>50%), and 79 due to severe hepatic dysfunction (Child–Pugh C).

Additionally, patients receiving dual antiplatelet therapy were excluded owing to the substantially elevated bleeding risk associated with combined antithrombotic regimens, which would confound the between-group comparison of DOAC-attributable bleeding. Patients who had undergone coronary angioplasty within the preceding 6 months were likewise excluded, as the mandatory post-procedural dual antiplatelet regimen in this population represents a distinct and dominant determinant of bleeding risk.

Patients with cancer-associated thrombosis or paraneoplastic thrombotic events were excluded (n = 231), because these conditions are associated with distinct bleeding risks and were analyzed separately in an independent cohort. In addition, extreme age ranges (<25 or >90 years; n = 147) were excluded to avoid disproportionate influence of outlier pharmacokinetic profiles and competing mortality risks. Three patients with documented hemarthroses were identified during screening; however, two were excluded due to active malignancy and one due to age above 90 years, in accordance with the predefined exclusion criteria, and therefore no patients with hemarthrosis were included in the final cohort.

The final study cohort comprised 5,721 eligible patients. All patients meeting inclusion criteria during the predefined study period were consecutively enrolled to minimize selection bias. Patients were followed from DOAC initiation to the first bleeding event, treatment discontinuation, death, or the end of the study period, whichever occurred first.

### Indications for oral anticoagulation

Indications for oral anticoagulation included non-valvular atrial fibrillation, venous thromboembolism (deep vein thrombosis and/or pulmonary embolism), postoperative prophylaxis after major orthopedic surgery, and other hypercoagulable states. Indications were classified according to the primary reason for DOAC initiation as documented in the electronic medical record.

### Endpoints and definitions

The primary endpoint was the occurrence of any bleeding event during follow-up, defined as a composite of major bleeding and clinically relevant non-major bleeding. Bleeding events were identified through structured review of electronic health records by two independent investigators using predefined case definitions. Major bleeding was categorized according to the International Society on Thrombosis and Haemostasis criteria, including fatal bleeding, symptomatic bleeding in a critical area or organ (e.g., intracranial, intraspinal, or intraocular), or bleeding causing a hemoglobin drop ≥2 g/dL or requiring transfusion of ≥2 units of blood. Clinically relevant non-major bleeding included epistaxis, bruising, gingival bleeding, hematuria, and gastrointestinal bleeding that did not meet criteria for major bleeding but resulted in unscheduled medical contact, interruption of therapy, or additional diagnostic evaluation. For patients with multiple bleeding episodes, only the first bleeding event was included in the analysis to avoid event clustering bias. This time-to-first-event approach was selected to avoid event clustering and to preserve the statistical independence of observations within the regression framework.

Secondary endpoints included life-threatening hemorrhagic events (intracranial hemorrhage, massive gastrointestinal bleeding, and hemorrhagic shock), non-hemorrhagic adverse reactions (particularly gastrointestinal intolerance and dyspepsia), and treatment discontinuation attributed to DOAC-related adverse effects.

### *In silico* analyses

Molecular docking was performed using AutoDock 4.2.6 (Morris et al., 2009) to investigate potential mechanistic differences among apixaban, dabigatran, edoxaban, and rivaroxaban. Docking protocols were validated by redocking the native ligand into the active site of the target protein to confirm reproducibility of the crystallographic binding pose. Receptor and ligand preparation, grid parameter definition, and docking execution were conducted within the AutoDockTools (MGLTools, version 1.5.6) environment.

At least 10 binding poses were generated for each protein-ligand pair using the Lamarckian Genetic Algorithm. The resulting conformations were evaluated according to binding energy (ΔG, kcal/mol), hydrogen bonding interactions, and root-mean-square deviation (RMSD). The pose with the lowest binding energy and an RMSD ≤ 2 Å was considered the most stable binding conformation.

Protein-ligand interactions and identification of hydrogen bonds were performed in the MGLTools environment. Additionally, three-dimensional (3D) protein-ligand complexes at the optimal docking positions were visualized using Python Molecular Viewer (PMW, version 1.5.6) (Sanner, 1999) software for visualization of molecular interactions.

#### Molecular docking study

In molecular docking analyses, three-dimensional (3D) crystal structures of human factor Xa and thrombin were retrieved from the Protein Data Bank (PDB; https://www.rcsb.org). For factor Xa, crystal structures complexed with apixaban (PDB ID: 2P16) (Pinto et al., 2007) and with rivaroxaban and edoxaban (PDB ID: 2W26) (Roehrig et al., 2005) were used. For thrombin, the crystal structure complexed with dabigatran (PDB ID: 1KTS) (Hauel et al., 2002) was selected. Docking simulations were performed using grid boxes centered on the active site and identical search parameters for all ligands to ensure comparability. Default AutoDock scoring functions were used, and the best-ranked binding pose based on binding energy was selected for analysis.

#### Preparation of receptor and ligand structures

The chemical structures of the ligands were generated using ChemDraw Ultra 12.0 (PerkinElmer Informatics. Waltham, MA, United States: PerkinElmer Informatics. Available from: https://www.perkinelmer.com). Ligand geometries were optimized in ChemBio3D Ultra 13.0 using the Merck Molecular Force Field 94 (MMFF94), and the lowest-energy conformations were retained (Halgren, 1996). The optimized structures were saved in PDB format and converted to PDBQT format Open Babel (version 3.1.1) for docking analysis (O’Boyle et al., 2011).

#### Molecular docking study with apixaban

The 3D structure of the protein (PDB ID: 2P16) was analyzed and found to consist of A and B chains. The structure was crystallized with a bound inhibitor ligand. Before docking, crystallographic water molecules were removed and polar hydrogen atoms were added. Appropriate partial charges were then assigned to prepare the receptor for docking. Molecular docking was performed after removal of the inhibitor ligand from the A chain using a grid box that encompassed the active site. The grid center coordinates were set to x = 7.5, y = 43.361, and z = 60.139 Å, with grid dimensions of 40 × 40 × 40 Å and a grid spacing of 0.375 Å.

#### Molecular docking study with rivaroxaban and edoxaban

The 3D structure of the protein (PDB ID: 2W26) was analyzed and found to consist of A and B chains. The structure was crystallized with a bound inhibitor ligand. Before docking, crystallographic water molecules were removed and polar hydrogen atoms were added. Molecular docking was performed after removal of the inhibitor ligand from the A chain using a grid box that encompassed the active site. The grid center coordinates were set to x = 8.917, y = 5.361, and z = 21.167 Å, with grid dimensions of 40 × 40 × 40 Å and a grid spacing of 0.375 Å.

#### Molecular docking study with dabigatran

The 3D structure of the protein (PDB ID: 1KTS) was analyzed and found to consist of A, B, and C chains. The structure was crystallized with a bound inhibitor ligand. Before docking, crystallographic water molecules were removed and polar hydrogen atoms were added. Molecular docking was performed after removal of the inhibitor ligand from the B chain using a grid box that encompassed the active site region. The grid center coordinates were set to x = 15.722, y = −15.556, and z = 23.944 Å, with grid dimensions of 40 × 40 × 40 Å and a grid spacing of 0.375 Å.

### Statistics

Statistical analyses were performed using IBM SPSS Statistics software, version 25.0 (IBM Corp., Armonk, NY, United States) (IBM Corp, 2017). Missing data were minimal and werehandled using complete-case analysis. Continuous variables were presented as mean ± standard deviation, and categorical variables as counts and percentages. Between-group comparisons among DOAC agents were performed using one-way analysis of variance (ANOVA) for continuous variables and chi-square tests for categorical variables. A multivariable logistic regression model was constructed to identify independent predictors of any bleeding. Clinically relevant covariates were selected *a priori* based on biological plausibility, established bleeding risk factors, and prior literature. These included age, sex, body mass index, estimated glomerular filtration rate (eGFR), prior bleeding, and the specific DOAC molecule. To preserve model stability and avoid multicollinearity, composite risk scores-including CHA_2_DS_2_-VASc and HAS-BLED-were not incorporated into the multivariable model, as these instruments subsume several of the individually entered covariates (age, renal function, prior bleeding history) and their simultaneous inclusion would introduce redundancy and unstable coefficient estimation. With respect to antiplatelet therapy, patients receiving dual antiplatelet regimens were excluded *a priori*; single antiplatelet use was distributed homogeneously across treatment groups and was therefore not retained as an independent covariate in the final model. Apixaban was chosen as the reference agent because of its widespread use and favorable bleeding profile in previous studies. Odds ratios (ORs) with 95% confidence intervals (CIs) were reported. Model assumptions, including absence of multicollinearity and adequacy of model fit, were assessed. Two-sided p values <0.05 were considered statistically significant.

## Results

### Baseline characteristics

Of the patients meeting the inclusion criteria (n = 5,721), 36.9% were prescribed rivaroxaban, 32.2% apixaban, 18.4% dabigatran, and 12.4% edoxaban (Table 1).

**TABLE 1:** Baseline demographic and clinical characteristics by DOAC agent.

Variable (n = 5,721)	Rivaroxaban (n = 2112)	Apixaban (n = 1846)	Dabigatran (n = 1054)	Edoxaban (n = 709)	SMD vs. Apixaban*
Clinical indications					
Atrial fibrillation (n, %)	1308 (61.9%)	1396 (75.6%)	602 (57.1%)	506 (71.4%)	0.293/ 0.382/ 0.093
Venous thromboembolism (n, %)	546 (25.9%)	264 (14.3%)	334 (31.7%)	70 (9.9%)	0.314/ 0.422/ 0.137
Other indications (n, %)	258 (12.2%)	186 (10.1%)	118 (11.2%)	133 (18.7%)	0.064/ 0.034/ 0.244
Demographic characteristics					
Mean age (years)	71.4 ± 9.2	74.1 ± 8.6	69.3 ± 10.4	75.8 ± 7.1	0.303/ 0.503/ 0.216
Female sex (%)	47.2	51.8	43.1	54.9	0.092/ 0.175/ 0.062
BMI (kg/m^2^)	28.6 ± 5.1	29.2 ± 4.8	27.3 ± 5.3	30.1 ± 4.4	0.121/ 0.376/ 0.195
Comorbidities					
Hypertension (%)	71.2	78.1	63.4	76.6	0.159/ 0.327/ 0.036
Diabetes mellitus (%)	29.7	34.2	22.1	36.5	0.097/ 0.272/ 0.048
Coronary artery disease (%)	46.1	49.7	38.2	51.4	0.072/ 0.233/ 0.034
Heart failure (%)	24.8	31.6	18.9	33.7	0.152/ 0.295/ 0.045
Prior stroke or TIA (%)	17.3	22.5	14.1	23.8	0.131/ 0.219/ 0.031
Peripheral artery disease (%)	9.6	12.4	7.2	13.8	0.090/ 0.176/ 0.042
Chronic liver disease (%)	3.1	3.8	2.4	4.1	0.038/ 0.081/ 0.015
History of prior bleeding (%)	8.9	11.7	6.2	12.9	0.092/ 0.194/ 0.037
Renal function					
eGFR <60 mL/min (%)	18.9	32.7	11.2	38.4	0.319/ 0.538/ 0.119
Concomitant therapy					
Chronic antiplatelet therapy (%)	26.5	29.8	21.7	30.6	0.073/ 0.186/ 0.017
Risk scores					
CHA_2_DS_2_-VASc score (mean ± SD)	3.6 ± 1.4	4.1 ± 1.3	3.2 ± 1.5	4.3 ± 1.2	0.370/ 0.641/ 0.160
HAS-BLED score (mean ± SD)	2.1 ± 0.9	2.4 ± 1.0	1.8 ± 0.8	2.5 ± 1.1	0.315/ 0.663/ 0.095

* Standardized mean differences were interpreted as follows: <0.10 negligible, 0.10–0.20 mild, and >0.20 clinically meaningful imbalance.

The distribution of clinical indications across treatment groups is presented in Table 1. Atrial fibrillation was the predominant indication in all groups, most frequently in apixaban-treated patients (75.6%), whereas venous thromboembolism was proportionally more common among dabigatran and rivaroxaban recipients. Baseline imbalances were most pronounced for age, renal dysfunction, CHA_2_DS_2_-VASc score, and HAS-BLED score, particularly between apixaban- and dabigatran-treated patients, as reflected by standardized mean differences exceeding 0.20.

Patients receiving apixaban and edoxaban were older on average and exhibited a higher prevalence of renal dysfunction, whereas dabigatran recipients tended to be younger and had fewer comorbid conditions. Women were slightly overrepresented in the apixaban and edoxaban groups. The prevalence of hypertension, diabetes mellitus, coronary artery disease, heart failure, prior stroke or TIA, and history of prior bleeding differed modestly among the four cohorts.

### Non–life-threatening complications

Non–life-threatening complications were common and demonstrated a distinct molecule specific pattern. Dabigatran was associated with a markedly higher prevalence of gastrointestinal intolerance and dyspeptic symptoms, whereas rivaroxaban and edoxaban were more frequently linked to bruising and minor mucosal bleeding manifestations, including gingival bleeding and prolonged minor bleeding. Edoxaban exhibited the highest rate of renal dose adjustment errors, consistent with its reliance on kidney function for appropriate dosing. When summarized as overall event burden, non–life-threatening adverse events were most frequent among dabigatran treated patients, occurring in 416 ± 5.1, patients (39.5%) followed by edoxaban with 254 ± 4.8 events (35.8%) and rivaroxaban with 711 ± 4.6 events (33.7%), whereas apixaban demonstrated the lowest cumulative burden with 495 events (26.8%) ± 3.9 (Table 2).

**TABLE 2:** Non–life-threatening complications by DOAC agent.

Complication	Rivaroxaban (n = 2112)	Apixaban (n = 1846)	Dabigatran (n = 1054)	Edoxaban (n = 709)
Gastrointestinal intolerance	133 (6.3%)	70 (3.8%)	133 (12.6%)	29 (4.1%)
Dyspepsia	68 (3.2%)	39 (2.1%)	103 (9.8%)	12 (1.7%)
Minor epistaxis	125 (5.9%)	79 (4.3%)	36 (3.4%)	33 (4.7%)
Bruising	150 (7.1%)	102 (5.5%)	41 (3.9%)	43 (6.1%)
Microscopic hematuria	59 (2.8%)	35 (1.9%)	25 (2.4%)	22 (3.1%)
Renal dose adjustment error	23 (1.1%)	15 (0.8%)	3 (0.3%)	35 (4.9%)
Gingival bleeding	93 (4.4%)	57 (3.1%)	28 (2.7%)	27 (3.8%)
Subconjunctival hemorrhage	44 (2.1%)	30 (1.6%)	13 (1.2%)	17 (2.4%)
Prolonged minor bleeding (>10 min)	78 (3.7%)	44 (2.4%)	22 (2.1%)	25 (3.5%)
Clinically relevant menorrhagia	38 (1.8%)	24 (1.3%)	12 (1.1%)	11 (1.6%)
Total number of adverse events	711 ± 4.6	495 ± 3.9	416 ± 5.1	254 ± 4.8

### Life-threatening hemorrhagic events

Life-threatening hemorrhagic events occurred in 3.1% of the overall cohort. Rivaroxaban and edoxaban were associated with numerically higher rates of severe gastrointestinal bleeding and intracranial hemorrhage, whereas apixaban demonstrated the lowest incidence across all life-threatening categories. Mortality directly attributable to bleeding events remained low but clinically meaningful (Table 3).

**TABLE 3:** Life-threatening adverse events by DOAC agent.

Complication	Rivaroxaban (n = 2112)	Apixaban (n = 1846)	Dabigatran (n = 1054)	Edoxaban (n = 709)
Intracranial hemorrhage	25 (1.2%)	7 (0.4%)	9 (0.9%)	9 (1.3%)
Massive GI bleeding	57 (2.7%)	20 (1.1%)	20 (1.9%)	16 (2.2%)
Trauma related major bleeding	14 (0.7%)	6 (0.3%)	4 (0.4%)	6 (0.8%)
Mortality attributed to event	13 (0.6%)	4 (0.2%)	4 (0.4%)	4 (0.5%)
Critical site bleeding (non-ICH)	17 (0.8%)	6 (0.3%)	5 (0.5%)	6 (0.9%)
Transfusion ≥4 units RBC	30 (1.4%)	11 (0.6%)	9 (0.9%)	9 (1.2%)
Emergency endoscopic/surgical hemostasis	40 (1.9%)	13 (0.7%)	12 (1.1%)	11 (1.6%)
ICU admission due to bleeding	23 (1.1%)	7 (0.4%)	6 (0.6%)	7 (1.0%)
Length of ICU stay (days, mean ± SD)	3.8 ± 1.6	3.1 ± 1.2	3.5 ± 1.4	3.9 ± 1.7
Total number of adverse events	219 ± 1.9	74 ± 1.2	69 ± 1.5	68 ± 1.7

When summarized as total life-threatening event burden, rivaroxaban accounted for 219 events (10.4%) ± 1.9, edoxaban for 68 events (9.6%) ± 1.7, dabigatran for 69 events (6.5%) ± 1.5, and apixaban for 74 events (4.0%) ± 1.2 (Table 3). Trauma related major bleeding constituted a small but clinically relevant proportion of these events. Given the relatively small number of events in the edoxaban group and the retrospective nature of the analysis, life-threatening hemorrhagic event rates should be interpreted as descriptive and exploratory rather than as definitive between-group comparisons.

### Any bleeding events and multivariable analysis

**FIGURE 1:**
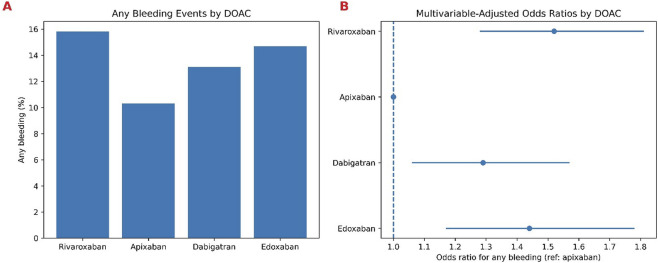
**(A)** Any bleeding events (%) according to DOAC agent. Bars indicate the proportion of patients experiencing any bleeding (major or clinically relevant non-major) in each treatment group. **(B)** Forest plot of multivariable-adjusted odds ratios for any bleeding by DOAC agent. Apixaban serves as the reference (OR 1.0). Points represent odds ratios; horizontal lines indicate 95% confidence intervals. The vertical dashed line marks the line of unity.

Any bleeding events (major or clinically relevant non-major) occurred in 13.5% of the overall cohort. Observed rates were 15.8% among rivaroxaban treated patients, 10.3% among apixaban treated patients, 13.1% among dabigatran treated patients, and 14.7% among edoxaban treated patients (Figure 1).

**FIGURE 2:**
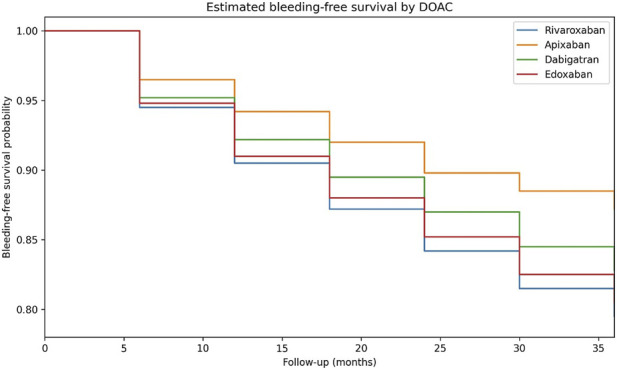
Estimated bleeding-free survival by DOAC agent over 36 months of follow-up. Curves represent time-to-event analysis estimates for the probability of remaining free from any bleeding event for each molecule.

In the multivariable logistic regression model, with apixaban as the reference agent, rivaroxaban (OR 1.52, 95% CI 1.28–1.81, p < 0.001), dabigatran (OR 1.29, 95% CI 1.06–1.57, p = 0.011), and edoxaban (OR 1.44, 95% CI 1.17–1.78, p = 0.001) were independently associated with higher odds of any bleeding. The absence of imbalance between treatment groups does not imply lack of predictive relevance of a variable. Additional independent predictors of bleeding included older age (per 10-year increment, OR 1.18, 95% CI 1.09–1.28, p < 0.001), impaired renal function defined as eGFR <60 mL/min (OR 1.37, 95% CI 1.19–1.58, p < 0.001), and prior bleeding history (OR 1.41, 95% CI 1.18–1.68, p < 0.001). Female sex was not significantly associated with bleeding risk (OR 1.08, 95% CI 0.95–1.22, p = 0.23). Estimated bleeding-free survival analysis suggested differences across treatment groups, with apixaban-treated patients appearing to have the highest event-free probability during follow-up, whereas rivaroxaban appeared to have the lowest bleeding-free survival, and dabigatran and edoxaban occupying intermediate positions (Figure 2).

### *In silico* findings

The interactions of apixaban, rivaroxaban, edoxaban, and dabigatran with target proteases (factor Xa and thrombin) were comparatively evaluated using AutoDock 4.2.6. Analyses performed over multiple binding poses allowed for consistent measurement of binding energy (ΔG), RMSD, and hydrogen bond networks, supporting the structural reliability of the selected poses. In analyses targeting Factor Xa, apixaban showed the lowest docking-derived binding free energy (ΔG = −14.46 kcal/mol) and formed stable hydrogen bond interactions with GLY216 and GLU146 within the active pocket.

**FIGURE 3:**
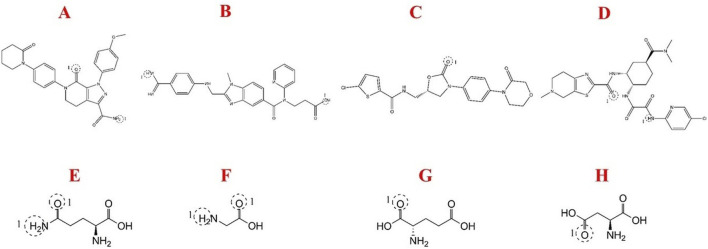
Two-dimensional schematic representation showing the interaction sites of apixaban, rivaroxaban, edoxaban, and dabigatran with critical amino acids to which they bind to different receptors. **(A)** Apixaban, **(B)** dabigatran, **(C)** rivaroxaban, **(D)** edoxaban, **(E)** glutamine (GLN), **(F)** glycine (GLY), **(G)** glutamic acid (GLU), and **(H)** aspartic acid (ASP).

**FIGURE 4:**
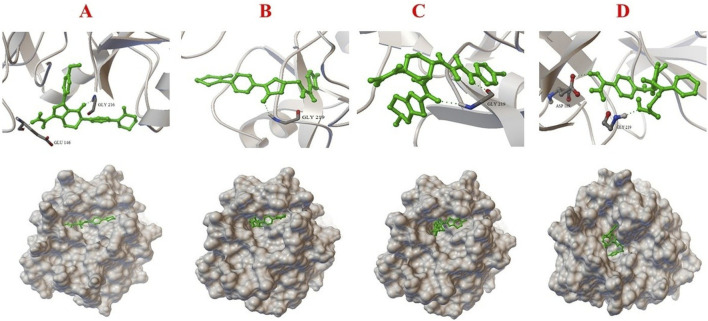
3D image of protein-ligand docking complex according to docking results. **(A)** Apixaban, **(B)** rivaroxaban, **(C)** edoxaban and **(D)** dabigatran.

Rivaroxaban (ΔG = −13.68 kcal/mol) and edoxaban (ΔG = −12.40 kcal/mol) showed lower affinities, with binding of both ligands predominantly concentrated around GLY219. In analyses of thrombin, the binding energy of dabigatran was calculated as −11.91 kcal/mol, and it was determined that it exhibits significant hydrogen bond interactions with GLY219 and ASP189 residues (Figures 3, 4; Table 4).

**TABLE 4:** Comparative molecular docking analysis of factor Xa and direct thrombin inhibitors.

Compound	Energy score Kcal/mol	RMSD	H-bond (distance Å)	PDB ID
Apixaban	−14.46	0.26	Apixaban O-1 with H-1 of GLY 216 (1.671) Apixaban H-1 with O-1 of GLU 146 (2.11)	2P16
Rivaroxaban	−13.68	0.22	Rivaroxaban O-1 with H-1 of GLY 219 (2.187)	2W26
Edoxaban	−12.40	0.33	Edoxaban O-1 with H-1 of GLY 219 (2.089) Edoxaban H-1 with O-1 of GLY 219 (1.957)	2W26
Dabigatran	−11.91	0.52	Dabigatran O-1 with H-1 of GLY 219 (2.013) Dabigatran H-1 with O-1 of ASP 189 (2.206)	1KTS

RMSD values for all ligands remained within 0.22–0.52 Å, indicating structurally consistent and reproducible binding conformations.

## Discussion

In this retrospective cohort, apixaban was associated with a comparatively lower risk of bleeding compared with other DOAC agents, while advanced age and renal dysfunction, emerged as independent predictors. These findings were interpreted alongside exploratory *in silico* analyses, which were intended to provide a hypothesis-generating framework to contextualize observed differences without implying causality.

While randomized controlled trials provide strong evidence regarding efficacy and safety, their highly selective patient populations may not fully reflect real-life practice (Blonde et al., 2018). Therefore, real-world data are becoming increasingly important in evaluating the comparative safety profiles of DOACs (Ma et al., 2025). Large observational studies and registry-based analyses have shown that apixaban may be associated with lower rates of major and intracranial hemorrhage compared to other DOACs; conversely, rivaroxaban and edoxaban carry a higher risk of bleeding in some subgroups, and dabigatran may be more disadvantageous in terms of gastrointestinal side effects (Lip et al., 2024). However, the biological mechanisms underlying these molecule-specific safety differences have not yet been fully elucidated.

The principal finding of this study is that, within this retrospective cohort, bleeding risk differed across individual DOAC molecules after adjustment for major clinical confounders. Although apixaban demonstrated relatively strong docking interactions with factor Xa, the absolute numerical differences in docking-derived parameters are modest and may fall within the expected variability of computational modeling and biological systems. However, these molecular findings should be regarded as exploratory and hypothesis-generating rather than as direct explanations of clinical outcomes.

It is important to recognize that plasma drug concentration, pharmacokinetics, and patient-specific variables remain the dominant determinants of anticoagulant effect *in vivo.* Prior pharmacokinetic studies have demonstrated a close linear relationship between anti-factor Xa activity and plasma apixaban concentrations across a wide dosing range (Hindley et al., 2023; Byon et al., 2019). Molecular interaction parameters alone are therefore not intended as primary clinical decision tools. Rather, docking-derived findings should be interpreted as hypothesis-generating mechanistic frameworks that may help contextualize observed clinical heterogeneity.

Enzyme inhibition *in vivo* depends on the fraction of unbound drug available at the target site over time, rather than solely on intrinsic binding affinity measured under idealized structural conditions. At first glance, the observation that apixaban demonstrated the lowest docking-derived binding free energy-suggesting stronger factor Xa affinity-may appear paradoxical in light of its lower observed bleeding rates. However, thermodynamic binding affinity derived from static *in silico* simulations does not directly determine the magnitude or duration of anticoagulant effect in patients. Clinical bleeding risk is primarily governed by systemic drug exposure, peak-to-trough plasma fluctuations, dosing frequency, renal clearance, and interindividual pharmacokinetic variability. In the case of apixaban, its twice-daily dosing regimen is associated with more stable plasma concentrations and attenuated peak (Cmax) excursions compared with once-daily factor Xa inhibitors. Consequently, even in the presence of strong molecular affinity, the absence of excessive peak-driven factor Xa suppression may limit bleeding propensity (Kim et al., 2018). Anticoagulant activity reflects a dynamic and reversible equilibrium between circulating free drug concentration and enzyme inhibition; therefore, docking-derived binding free energy cannot be interpreted as a surrogate for sustained *in vivo* enzyme occupancy or cumulative anticoagulant intensity. These considerations help reconcile the apparent discrepancy between stronger docking affinity and lower observed clinical bleeding incidence.

The present analysis demonstrates that the safety profiles of direct oral anticoagulants are molecule-specific rather than class-uniform. While prior randomized trials established the efficacy of DOACs versus VKAs, real-world safety differences among individual agents remain incompletely understood. Our study contributes to this area by integrating real-world bleeding phenotyping with molecular docking analyses, offering a framework that may help contextualize observed safety heterogeneity. In our cohort, apixaban was consistently associated with the lowest incidence of overall and life-threatening bleeding, even after multivariable adjustment for age, renal function, and other established clinical risk factors. These findings align with recent real-world and pharmacovigilance studies reporting a more favorable bleeding profile for apixaban compared with other DOACs, particularly rivaroxaban and edoxaban (Qian et al., 2024; Wu et al., 2022; Jeong et al., 2024).

Importantly, our study complements existing clinical observations by providing a mechanistic context through molecular docking analyses. In our *in silico* model, apixaban demonstrated the lowest docking-derived binding free energy toward factor Xa and formed stable hydrogen bond interactions within the active site.

Such stable drug–target interactions should not be interpreted as clinically meaningful pharmacodynamic determinants, but rather as comparative structural features observed in computational modeling; however, clinical bleeding risk is primarily determined by patient-level and pharmacokinetic factors (Hindley et al., 2023). In contrast, rivaroxaban and edoxaban demonstrated higher (less negative) docking-derived binding energies and more localized interaction patterns, findings that may provide a potential mechanistic context for their comparatively higher bleeding rates reported in clinical practice (Qian et al., 2024; Wu et al., 2022). Dabigatran, as a direct thrombin inhibitor, exhibited selective but relatively weaker docking interactions with thrombin. Clinically, this agent has been associated with greater gastrointestinal intolerance and treatment discontinuation, observations that are broadly consistent with recent mechanistic and clinical studies highlighting thrombin–PAR signaling and gastrointestinal vulnerability (Dólleman et al., 2022; Wu et al., 2022; Chen et al., 2023).

The numerical differences in docking energies between factor Xa inhibitors are relatively modest and should be interpreted cautiously, as variations of this magnitude may fall within the expected uncertainty of computational modeling and biological variability.

Several prior investigations have examined the relationship between pharmacokinetic–pharmacodynamic properties of direct oral anticoagulants and their clinical safety profiles. While most studies interpret safety differences primarily through clinical and pharmacokinetic variables, experimental enzymatic studies have reported subnanomolar inhibition constants Ki (Ki ≈ 0.08 nM) and dissociation parameters for apixaban–factor Xa interactions, indicating very high target affinity, as described by Pinto et al. (2007). Although docking-derived binding energies cannot be numerically equated with experimental Ki or Kd values, the comparatively lower ΔG observed for apixaban in our simulations is directionally consistent with its experimentally demonstrated high affinity. This correspondence should be interpreted as qualitative and hypothesis-supporting rather than quantitative agreement.

By integrating docking-derived binding characteristics with large-scale real-world outcomes, our results support a precision-oriented approach to anticoagulant selection. Contemporary literature increasingly emphasizes individualized DOAC choice based on patient-specific bleeding risk and drug-specific pharmacology rather than interchangeability within the class (Hindley et al., 2023; Qian et al., 2024; Wu et al., 2022).

In addition to real-world evidence, pivotal randomized controlled trials provide an essential benchmark for interpreting the present findings. In the ARISTOTLE trial, apixaban demonstrated a significantly lower rate of major bleeding and intracranial hemorrhage compared with warfarin, establishing its favorable safety profile (Granger et al., 2011). In contrast, the ROCKET-AF trial reported higher rates of gastrointestinal bleeding with rivaroxaban (Patel et al., 2011), while the ENGAGE AF–TIMI 48 trial showed dose-dependent bleeding outcomes with edoxaban (Giugliano et al., 2013). Although these trials were not designed for direct head-to-head DOAC comparisons, their bleeding patterns broadly parallel observed in our real-world cohort (Lawal et al., 2025). When interpreted alongside contemporary systematic reviews and mechanistic pharmacokinetic–pharmacodynamic analyses, these RCT data reinforce the concept that molecule-specific interaction kinetics with factor Xa contribute to clinically meaningful safety differences among DOACs (Byon et al., 2019).

The molecular docking analyses performed to support these clinical observations provide a hypothesis-generating mechanistic context to support the interpretation of observed clinical safety differences, rather than a mechanistic explanation of causality.

In the *in silico* evaluations conducted using AutoDock 4.2.6, apixaban demonstrated the strongest binding affinity to factor Xa (ΔG = −14.46 kcal/mol) and formed more stable and directionally oriented hydrogen bond networks between GLY216 and GLU146 within the active site. In contrast, rivaroxaban (−13.68 kcal/mol) and edoxaban (−12.40 kcal/mol) exhibited weaker binding energies, with their interactions predominantly localized around a single residue (GLY219). The direct thrombin inhibitor dabigatran displayed a comparatively lower binding energy (−11.91 kcal/mol) and a limited hydrogen bond network.

Taken together, the convergence of clinical and *in silico* findings permits the formulation of a testable mechanistic hypothesis: DOACs exhibiting more favorable active-site interaction geometry-as reflected by lower docking-derived binding free energies and multi-residue hydrogen bond networks-may engage their target protease in a structurally more stable manner, potentially contributing to more predictable pharmacodynamic behavior. In the case of apixaban, stronger and more directionally oriented binding interactions with GLY216 and GLU146 within the factor Xa active site may correspond, at least in part, to the attenuated peak-to-trough anticoagulant fluctuation observed pharmacokinetically with twice-daily dosing. In contrast, rivaroxaban and edoxaban, which demonstrated more localized single-residue interactions and higher binding energies, were associated with higher observed bleeding rates in this cohort-a pattern that may be explored prospectively in mechanistic studies incorporating pharmacokinetic-pharmacodynamic modeling. Dabigatran’s selective thrombin inhibition with a comparatively lower binding energy aligns with its distinct clinical profile, characterized by greater gastrointestinal vulnerability and thrombin–PAR1 signaling-related effects rather than hemorrhagic dominance.

These findings do not imply a causal relationship between docking-derived binding properties and clinical bleeding outcomes. Instead, the *in silico* results are presented as hypothesis-generating, structure-based observations that may provide mechanistic context to the real-world safety patterns observed in this cohort. Clinical outcomes remain primarily driven by pharmacokinetic properties, patient-specific factors, and real-world treatment conditions, with molecular modeling serving only as supportive mechanistic interpretation.

Real-world comparative analyses have reported lower major bleeding rates with apixaban compared with rivaroxaban and dabigatran in patients with nonvalvular atrial fibrillation (Noseworthy et al., 2016; Pozzi et al., 2024). The results of the current study are concordant with these contemporary reports and extend them by integrating supportive *in silico* mechanistic data, thereby providing a hypothesis-generating, clinically anchored interpretative framework rather than a biological explanation for the observed clinical differences.

From a clinical standpoint, these findings reinforce the principle that selection among DOAC agents should account for molecule specific safety profiles rather than presuming full therapeutic interchangeability. In routine practice, anticoagulant choice is primarily guided by patient characteristics, comorbidity burden, renal function, bleeding history, and the strength of evidence from randomized trials and guideline recommendations. Within this context, insights derived from molecular interaction analyses may provide additional explanatory value when interpreting differences in bleeding susceptibility observed across agents. Such an approach is intended to complement, not supplant, evidence-based practice and individualized clinical judgment.

From a practical clinical standpoint, standard coagulation assays — including prothrombin time and activated partial thromboplastin time — do not reliably quantify the anticoagulant effect of DOACs and should not be used for therapeutic monitoring. Agent-specific assays are required when quantification is clinically necessary: calibrated anti-factor Xa chromogenic assays provide the most reliable estimates of on-therapy drug exposure for apixaban, rivaroxaban, and edoxaban, whereas diluted thrombin time (dTT; Hemoclot assay) or the ecarin clotting time (ECT) are the preferred methods for dabigatran quantification (Pozzi et al., 2024). In the setting of life-threatening hemorrhage or urgent surgical intervention, rapid reversal of anticoagulant effect is essential. Idarucizumab, a humanized monoclonal antibody fragment, provides immediate and complete neutralization of dabigatran. For factor Xa inhibitors, andexanet alfa offers targeted reversal by competitively binding free drug, while four-factor prothrombin complex concentrates represent an alternative when andexanet alfa is unavailable or contraindicated (Pozzi et al., 2024; Muir et al., 2024).

This study has several limitations. First, its retrospective observational design precludes causal infer ence and is subject to residual confounding despite multivariable adjustment. Additionally, the time-to-first-event design does not capture recurrent bleeding events or subsequent complications following an initial bleeding episode, which should be considered when interpreting the results. In addition, differences in the distribution of clinical indications across treatment groups may have contributed to the observed outcomes and should be considered as a potential source of residual confounding. Furthermore, although composite risk scores and select concomitant therapies were not incorporated into the multivariable model, this decision was informed by considerations of multicollinearity and model stability rather than their clinical irrelevance; residual confounding from unmeasured or incompletely adjusted variables cannot be excluded. Second, bleeding events were identified from electronic health records and may underestimate minor events that did not result in healthcare contact. Third, the single-center nature of the cohort may limit generalizability to other healthcare settings. With respect to the *in silico* analyses, molecular docking represents a static approximation of drug–target interactions and does not capture dynamic conformational changes, solvent effects, or *in vivo* pharmacokinetic variability. Accordingly, docking-derived binding energies should be interpreted as comparative rather than absolute measures. From a hypothesis-generating perspective, these findings suggest that differences in molecular binding profiles among DOACs may contribute to variability in bleeding risk, warranting further mechanistic and prospective investigation. Moreover, docking simulations do not account for important clinical determinants of bleeding risk, including plasma protein binding, metabolite activity, and tissue distribution (Hindley et al., 2023). In addition, Betrixaban was not represented in the cohort because it was not available for clinical use in the country where the study was conducted during the data collection period.

## Conclusion

Within this retrospective cohort, apixaban was associated with the lowest observed bleeding risk among the four DOAC agents examined, with rivaroxaban, edoxaban, and dabigatran each demonstrating independently higher odds of bleeding after multivariable adjustment. Advanced age and renal dysfunction emerged as additional patient-level determinants of hemorrhagic risk, consistent with established pharmacokinetic vulnerability profiles of this drug class. The molecular docking analyses, conducted as a complementary and hypothesis-generating component of this study, demonstrated that apixaban exhibits the most favorable active-site interaction geometry among factor Xa inhibitors, characterized by lower binding free energy and a multi-residue hydrogen bond network. These structural observations, taken together with the clinical findings, permit the formulation of a testable mechanistic hypothesis: that more stable and geometrically oriented drug–target engagement may contribute to more predictable pharmacodynamic behavior, potentially manifesting as attenuated peak-driven anticoagulant fluctuation and reduced hemorrhagic propensity. This hypothesis requires prospective mechanistic validation and should not be construed as a causal explanation of the observed clinical differences. Rivaroxaban, edoxaban, and dabigatran remain clinically appropriate agents in carefully selected patient phenotypes, particularly where renal function trajectories, dosing interval considerations, or cancer-associated thrombosis alter the therapeutic calculus. DOAC selection should remain individualized, integrating patient-specific bleeding diathesis, comorbidity burden, and the weight of randomized trial evidence rather than molecular interaction parameters alone. Collectively, these findings contribute to the emerging framework of precision anticoagulation, in which molecule-specific pharmacodynamic characteristics are considered alongside clinical risk stratification in therapeutic decision-making.

## Data Availability

The original contributions presented in the study are included in the article/supplementary material, further inquiries can be directed to the corresponding author.
